# In-Line Measurement of Water Contents in Ethanol Using a Zeolite-Coated Quartz Crystal Microbalance

**DOI:** 10.3390/s151027273

**Published:** 2015-10-27

**Authors:** Byoung Chul Kim, Takuji Yamamoto, Young Han Kim

**Affiliations:** 1 Department of Advanced Materials and Chemical Engineering, Kyungnam College of Information & Technology, Pusan 617-701, Korea; E-Mail: kimbc@eagle.kit.ac.kr; 2 Department of Chemical Engineering, University of Hyogo, Himeji 671-2201, Japan; E-Mail: tyamamot@eng.u-hyogo.ac.jp; 3 Department of Chemical Engineering, Dong-A University, Pusan 604-714, Korea

**Keywords:** quartz crystal microbalance, ethanol dehydration, zeolite-coated sensor

## Abstract

A quartz crystal microbalance (QCM) was utilized to measure the water content in ethanol. For the improvement of measurement sensitivity, the QCM was modified by applying zeolite particles on the surface with poly(methyl methacrylate) (PMMA) binder. The measurement performance was examined with ethanol of 1% to 5% water content in circulation. The experimental results showed that the frequency drop of the QCM was related with the water content though there was some deviation. The sensitivity of the zeolite-coated QCM was sufficient to be implemented in water content determination, and a higher ratio of silicon to aluminum in the molecular structure of the zeolite gave better performance. The coated surface was inspected by microscopy to show the distribution of zeolite particles and PMMA spread.

## 1. Introduction

Ethanol becomes a typical azeotropic mixture with water at a concentration of 96%, which makes producing pure ethanol difficult [1]. Instead of common distillation, a variety of entrainers are utilized in extractive distillation processes to yield highly concentrated ethanol. In-line monitoring of the water content in ethanol is important for the operation of the concentration process. In particular bioethanol production from grain fermentation relies heavily on ethanol distillation. The in-line measurement of water content is necessary for the control of the distillation column by adjusting steam and cooling water flows to the column. Recently the in-line measurement has been applied to monitor water content in food processing [2], coating processes [3] and cement hydration [4].

An off-line technique for measuring the water content from the distillate volume fractions was introduced, where a standard distillation apparatus was used [5]. As an in-line application, Fujiwara *et al.* [6] determined the liquid density by measuring the reflection intensity of the applied laser light, and calculated the water content. The absorbance of near IR in the liquid sample was also measured to determine the water content [7]. Instead of light variation, ultrasound waves were applied to measure its propagation velocity, and the water content was calculated from the density difference detected by the variation of wave velocity in commercial beverages [8]. High frequency electric signals have been used for water content measurement in liquid samples [9,10] and leaves [11]. The dielectric permittivity was measured from the variation of amplitude to determine the water content. Other electric sensors measuring conductivity were introduced for various applications of water content measurement in liquids and solids [12] and fuel ethanol [13].

Though the measurement of electro-conductivity in ethanol is simple, the arrangement of the measuring cell and the liquid sample flow affect the measurement results. Because the liquid flow on the cell surface changes, the conductivity variation explains the change of liquid flow. The liquid flow on the electrode surface was used for the study of flow and transient behavior of streams [14]. A quartz crystal microbalance (QCM) has a thin quartz plate in the middle, and it has two electrodes on both sides of the plate. When an alternating electric field is applied across the quartz plate, it causes the vibration of the plate and the vibration leads to a resonant oscillation in the electric field. The vibration frequency is called resonant frequency of the quartz plate, and is sensitive to the changes of loading and the micro-rheology on the electrode. The QCM application uses the frequency variation to detect the changed loading. The variation of mass loading was employed to detect the dew point of organic vapor condensing on an electrode surface [15]. The variation of micro-rheology was also used to determine the kinetic rate of photopolymerization [16,17] and the variation of the thermal properties of polymers [18].

When the collection of target material on the electrode is difficult, the electrode surface is modified to enhance the loading. In the determination of organic substances either in gas or liquid phase, various organic films are coated on the surface to measure substances in gas and liquid form [19,20,21,22,23,24,25], and photonic film immobilization has been developed for the coating [26]. The water content in ethanol has been measured using a polyvinyl alcohol (PVA)-coated QCM [27]. Zeolites are good adsorbents for many different applications in gas and liquid systems. Especially the pore size in zeolites is manipulated for special purposes, and the zeolite used here is designed for dehydration of liquid materials, whereby the zeolite adsorbs the water in ethanol to increase its own mass, of which the variation is detected with the QCM. The location of water molecules and their interactions with the zeolite framework and extra-framework cations have been the subject of many experimental studies [28,29]. In this study, a measurement device for water content in ethanol is developed by applying zeolite particles on the electrode surface of the QCM. Its performance is examined with 1% to 5% water in ethanol. The characteristics of the coating were examined by microscopic observation.

## 2. Experimental Section

### 2.1. Materials

The five kinds of zeolite used were from Wako Chemical (Osaka, Japan), and ACS Grade ethyl alcohol of 99.98% purity and 0.007% water content was from Burdick & Jackson (Ulsan, Korea). No detectable entrainer was present in the ethyl alcohol. Acetone was from Junsei Chemical (Tokyo, Japan), and poly(methyl methacrylate) (PMMA) was obtained from Aldrich Chemical (St. Louis, MO, USA). The chemicals were used as received. The quartz crystal microbalance (Sunny Electronics Co., Chungju, Korea) was capped for electronic circuit use and purchased from a local store. The cap was removed before coating the zeolite particles.

### 2.2. Instruments

Microscopic observations were done with a scanning electron microscope (Model JSM 6700F, JEOL Ltd., Tokyo, Japan) and an optical microscope (Sometech, Model iMegascope, Seoul, Korea). A spin coater (Model ACE-1020S, Dong Ah, Korea) was used for binder coating. When an oscillation circuit using a NAND gate TTL IC is applied to the QCM, an electric wave of continuous oscillation is generated between two conductors of the QCM. The oscillation circuit was assembled at the lab. The frequency of the continuous oscillation was measured with a home-made frequency counter with an IC counter (ICM 7216, Intersil, Milpitas, CA, USA). The counter was connected with a PC to collect the measurement data.

### 2.3. Equipment

An AT-cut quartz crystal microbalance having a base frequency of 8 MHz was utilized in the experiments. The electrodes of the QCM were silver finished on the unpolished surface of the quartz plate. Five kinds of zeolite were coated on the electrode, after PMMA binder was spread on it.

**Figure 1:**
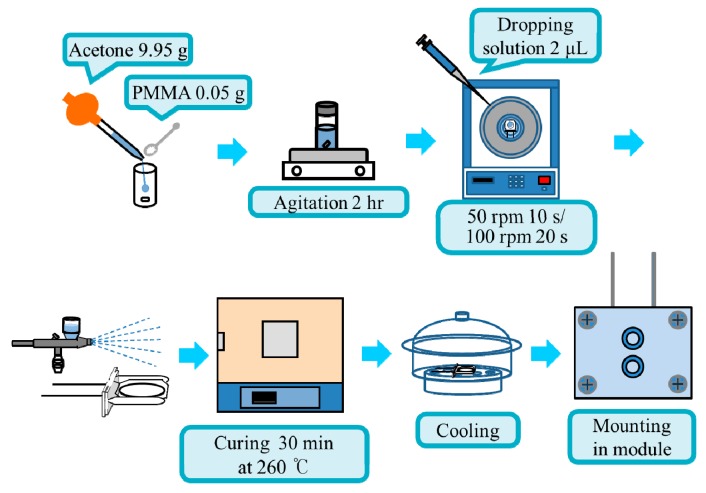
Flowchart of the preparation procedure of zeolite-coated quartz crystal microbalance.

The PMMA (0.05 g) was dissolved in acetone (9.95 g) in a beaker, and the mixture was agitated for 2 h for complete dissolution. Because the top plate cannot hold the QCM, a specially designed silicone rubber holder was utilized in the spin coating. A QCM was placed in the holder, and the holder was set in the center of the top plate of the spin coater. When the coater was ready, the PMMA solution (2 μL) was dropped on the electrode and the coater was activated. The spinning was for 10 s at 50 rpm followed by 20 s at 100 rpm. After the binder was spread on the electrode, the zeolite powder was sprayed on the electrode using an air brush with mild air flow. The coated resonator was cured in an oven of 260 °C for 30 min to make the powder bind on the electrode. The QCM preparation procedure is illustrated in Figure 1. After cooling in a desiccator, the QCM was mounted in the cell module.

**Figure 2:**
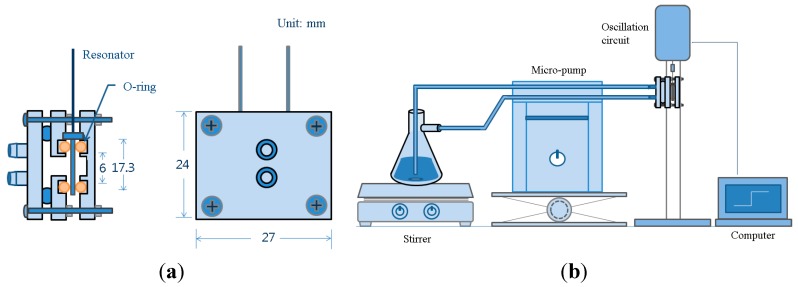
Schematic diagram of the cell module (**a**) Cell configuration; (**b**) Experimental setup for water content measurements.

The cell module holding the QCM was built with polypropylene plates as shown in Figure 2a. The thickness of three plates was 5 mm. There are two cells—one for the ethanol sample and the other for QCM mounting. The leftmost plate has two nipples for ethanol flow. Three o-rings were placed between the plates avoid ethanol leakage. The size of the plates was 27 mm by 24 mm. Four screws tightened the plates.

### 2.4. Procedures

The zeolite-coated QCM was mounted in the cell module, and its two conductors were connected to the oscillation circuit as illustrated in Figure 2b. While the known concentration of water in ethanol was supplied at a rate of 16 mL/min, the resonant frequency was measured continuously. Due to the size limitation of the QCM module a large amount of sample flow is not allowed, but taking a small amount of sample from the process line and returning the sample to the line provide the in-line measurement of the water content in the industrial process of ethanol concentration. The oscillation circuit provides a small amount of oscillating current to the QCM, and the resonant frequency is determined by the load variation on the surface of the QCM electrode. The frequency is counted with a home-made counter connected to a PC. The measured frequency was stored in the PC for later data analysis. When the measured frequency is stable, water is added to ethanol to increase the water content by 1% steps each time up to 5%. The same procedure was applied to the next QCM for the measurement.

## 3. Results and Discussion

Five kinds of synthetic zeolite were utilized to prepare the zeolite-coated QCM as described above, and their detection performance of water content in ethanol was examined here. The characteristic properties of the zeolites, including adsorption surface area and pore volume, are listed in Table 1.

**Table 1:** Characteristics of zeolite used in the experiments including silicon to aluminum ratio, Brunauer-Emmett-Teller (BET) area and pore volume.

Zeolite	Formula	Si/Al Ratio	BET Area (m^2^/g)	Pore Volume (cm^3^/g)
A-3	(1-X)Na_2_O·XK_2_O·Al_2_O_3_·2SiO_2_ (X > 0.4)	1	–	–
A-5	(1-X)Na_2_O·XK_2_O·Al_2_O_3_·2SiO_2_ (X > 0.7)	1	600	0.3
Na-Y	Na_2_O·Al_2_O_3_·5.5SiO_2_	11	810	0.4
Na-MOR	Na_2_O·Al_2_O_3_·18SiO_2_	36	470	0.23
H-MOR	0.12Na_2_O·Al_2_O_3_·240SiO_2_	400	550	0.25

**Figure 3:**
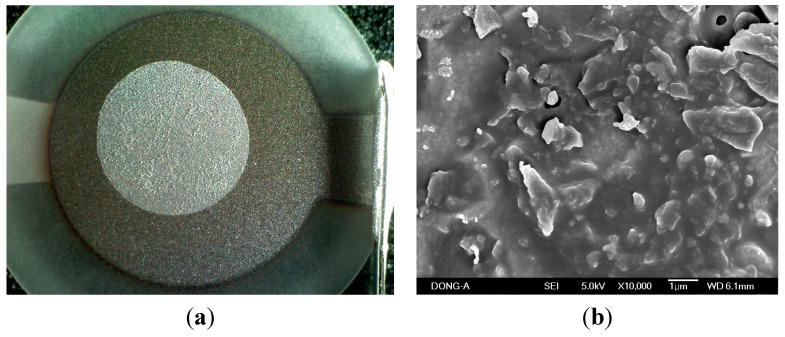
Photographs of the QCM surface by (**a**) Optical microscope; (**b**) Scanning electron microscope (×10,000 times). In image (a), the outer circle with connection path on the right-hand side is the silver electrode, and white inner circle demonstrates the PMMA coating. The center of the QCM is more sensitive than the outer area.

For the inspection of the zeolite coating on the QCM surface, microscopic observations were conducted (Figure 3).

The observation with an optical microscope is shown in Figure 3a. The white circle on the electrode indicates the PMMA film, and fine particles are the zeolite particles spread on the film. When the frequency drop from the 8 MHz base frequency of the QCM is larger than 20,000 Hz due to the coating of PMMA and zeolite, the QCM stops oscillating. Considering the amount of PMMA used as a binder in the zeolite coating, the amount of zeolite was adjusted for the frequency drop of around 5000 Hz. The magnified picture of the zeolite-coated QCM exhibited in Figure 3b was taken with the SEM. A portion of binder melted at the curing temperature of 260 °C, and some of the zeolite particles were covered by the binder. Though the binder covered the zeolite particles in the coating process, the pores of the zeolite particles were reactivated for the adsorption of water molecule during the curing at 260 °C.

**Figure 4:**
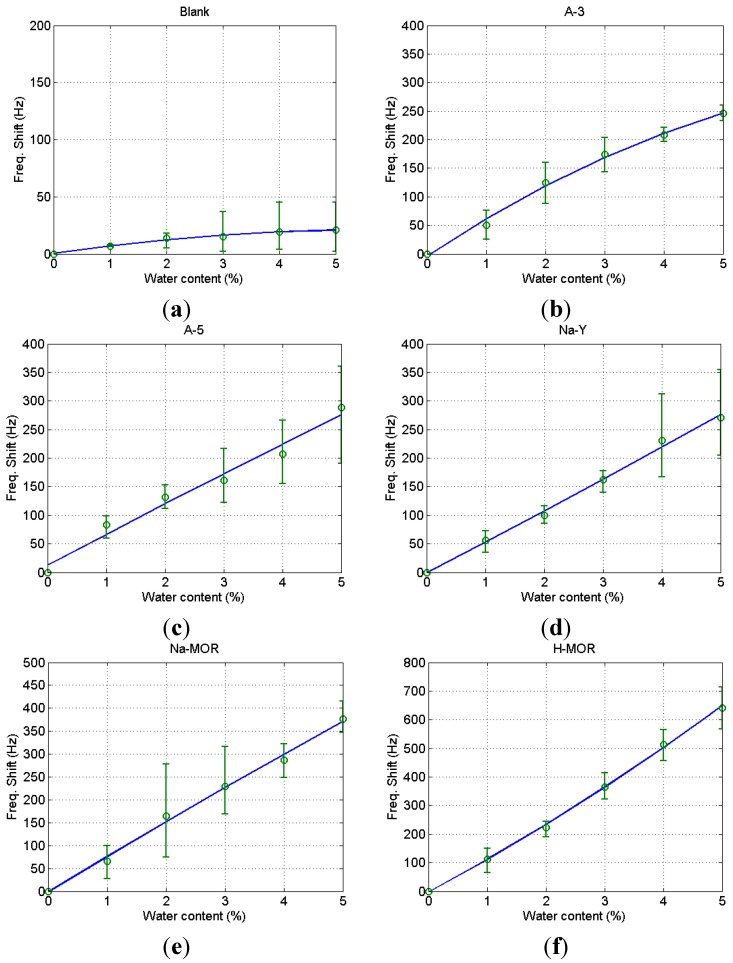
Frequency shifts of blank QCM (**a**) A-3 coated QCM; (**b**) A-5 coated QCM; (**c**) Na-Y coated QCM; (**d**) Na-MOR coated QCM; (**e**) H-MOR coated QCM; (**f**) At different water contents.

To examine the effect of the bare QCM and binder on the water adsorption in the zeolites, blank tests were conducted with the PMMA-only coated QCM. Figure 4a shows the frequency drop with the increase of water content in ethanol. The line connecting the circles indicates the 2nd order fitted curve of the experimental data. The average values of drop of the blank QCM at a given water content were deduced from the measurements of the zeolite-coated QCM. Because the zeolite used in the experiments was commercially developed for the use in ethanol dehydration, potential contaminants in the ethanol of commercial production do not affect the water adsorption performance in this study. The water adsorption with the A-3 coated QCM is shown in Figure 4b. The rigid attachment of zeolite of mass change to the crystal surface due to the adsorption of water causes a decrease of frequency in the resonant frequency [30]. The increase of frequency drop with the elevation of water content indicates that the zeolite coating works for the detection of water content in ethanol. Though the variation of measurements was slightly large at low and middle water contents, less variation was observed at high contents. Contrarily the variation of measurements was large with high contents in the measurement with the A-5 coated QCM as illustrated in Figure 4c. The amount of variation at a certain water content, the sensitivity, is similar for the A-3 coated and A-5 coated QCMs. A similar measurement of frequency drop was yielded with the Na-Y coated QCM as plotted in Figure 4d, but large variation was observed at high content of water. An improvement of sensitivity was obtained with the Na-MOR coated QCM as demonstrated in Figure 4e. About 37% of the sensitivity improvement was found compared to the previous three QCMs. The best performance was yielded with the H-MOR coated QCM, in which almost 230% of improvement was achieved over the first three QCMs as shown in Figure 4f. When the performance improvement was compared to the molecular composition of the zeolite, it was found that the molecular ratio of silicon to aluminum affects the sensitivity of water adsorption. The highest ratio of silicon in H-MOR gives the best performance, while the adsorption surface area and pore volume do not affect the sensitivity of the water detection. There was some deviation in the measurement of water content in ethanol using the zeolite-coated QCM, but all five kinds of zeolites showed an increase in frequency drop as the water content increased. Especially the H-MOR coated QCM lead to the highest sensitivity with small variation in the measurement. The best limit of detection (LOD) was obtained from the H-MOR, which was 0.1% considering the variance of the measurement. This limit of concentration measurement is considered acceptable for process control use in industrial applications.

**Figure 5:**
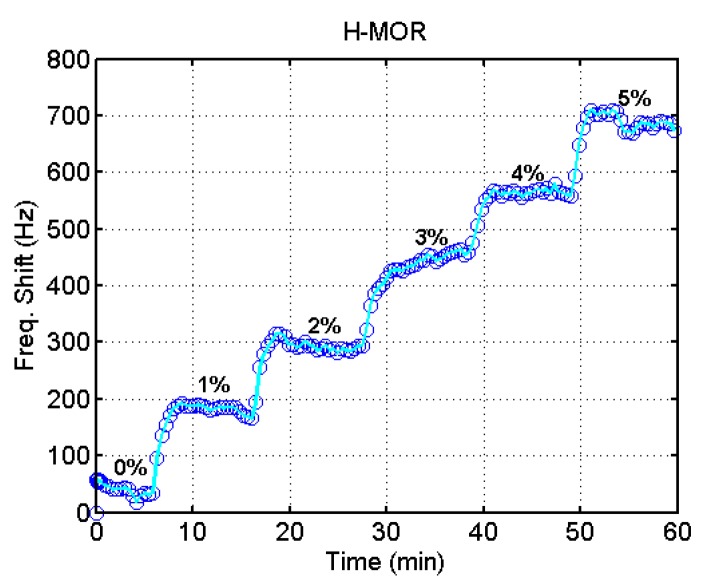
Temporal variation of frequency shift of the H-MOR coated QCM at different water contents.

**Figure 6:**
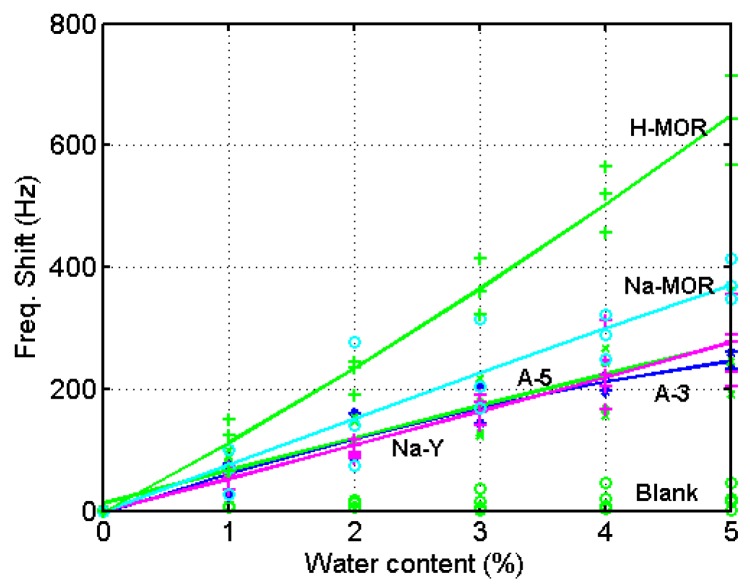
Comparison of frequency shifts among different zeolite coatings.

Figure 5 shows the temporal variation of frequency shift of the H-MOR coated QCM at different water contents. Though there is baseline drift in the measurements, the increase of water content gives a significant change of the frequency shift. For comparison among the different zeolites, the frequency shift of all QCMs used in this study is displayed in Figure 6.

## 4. Conclusions/Outlook

A simple device using a quartz crystal microbalance (QCM) was proposed to measure water content in ethanol. The QCM was coated with zeolite particles and PMMA thin film as a binder for improved water content measurement sensitivity, and its performance was investigated in a flowing stream of ethanol with known water content. The experimental results indicate that the zeolite coating improves the detection sensitivity, and the higher ratio of silicon to aluminum in the molecular structure of zeolite gives higher sensitivity. Though there was some deviation in the measurements, the higher water content in ethanol results in more drop of resonant frequency of the QCM. The relation between the content and frequency drop provides the determination of water content in unknown samples. The microscopic observation of the QCM surface displayed the distribution of the zeolite particles on the surface.
